# Supplementary material for: Iterative improvement in the automatic modular design of robot swarms
Source: PeerJ Comput Sci. 2020 Dec 7;6:e322. doi: 10.7717/peerj-cs.322 (PMC7924708; doi:10.7717/peerj-cs.322)
Supplement: Supplemental Information 3 [file peerj-cs-06-322-s003.zip › argos3/doc/api/standalone/a00572_source.html]

ARGoS: plugins/simulator/entities/led\_entity.cpp Source File


- Main Page
- Related Pages
- Namespaces
- Classes
- Files

- File List
- File Members

# plugins/simulator/entities/led\_entity.cpp

Go to the documentation of this file.

```
00001 
00007 #include "led_entity.h"
00008 #include <argos3/core/simulator/simulator.h>
00009 #include <argos3/core/simulator/space/space.h>
00010 #include <argos3/plugins/simulator/media/led_medium.h>
00011 
00012 namespace argos {
00013 
00014    /****************************************/
00015    /****************************************/
00016 
00017    CLEDEntity::CLEDEntity(CComposableEntity* pc_parent) :
00018       CPositionalEntity(pc_parent),
00019       m_pcMedium(NULL) {
00020       Disable();
00021    }
00022 
00023    /****************************************/
00024    /****************************************/
00025 
00026    CLEDEntity::CLEDEntity(CComposableEntity* pc_parent,
00027                           const std::string& str_id,
00028                           const CVector3& c_position,
00029                           const CColor& c_color) :
00030       CPositionalEntity(pc_parent, str_id, c_position, CQuaternion()),
00031       m_cColor(c_color),
00032       m_cInitColor(c_color),
00033       m_pcMedium(NULL) {
00034       SetColor(c_color);
00035    }
00036 
00037    /****************************************/
00038    /****************************************/
00039 
00040    void CLEDEntity::Init(TConfigurationNode& t_tree) {
00041       try {
00042          /* Parse XML */
00043          CPositionalEntity::Init(t_tree);
00044          GetNodeAttribute(t_tree, "color", m_cInitColor);
00045          m_cColor = m_cInitColor;
00046       }
00047       catch(CARGoSException& ex) {
00048          THROW_ARGOSEXCEPTION_NESTED("Error while initializing led entity", ex);
00049       }
00050    }
00051 
00052    /****************************************/
00053    /****************************************/
00054 
00055    void CLEDEntity::Reset() {
00056       m_cColor = m_cInitColor;
00057    }
00058 
00059    /****************************************/
00060    /****************************************/
00061 
00062    void CLEDEntity::Destroy() {
00063       if(HasMedium()) {
00064          RemoveFromMedium();
00065       }
00066    }
00067 
00068    /****************************************/
00069    /****************************************/
00070 
00071    void CLEDEntity::SetEnabled(bool b_enabled) {
00072       CEntity::SetEnabled(m_cColor != CColor::BLACK);
00073    }
00074 
00075    /****************************************/
00076    /****************************************/
00077 
00078    void CLEDEntity::SetColor(const CColor& c_color) {
00079       m_cColor = c_color;
00080       SetEnabled(c_color != CColor::BLACK);
00081    }
00082 
00083    /****************************************/
00084    /****************************************/
00085 
00086    void CLEDEntity::AddToMedium(CLEDMedium& c_medium) {
00087       if(HasMedium()) RemoveFromMedium();
00088       m_pcMedium = &c_medium;
00089       c_medium.AddEntity(*this);
00090       Enable();
00091    }
00092 
00093    /****************************************/
00094    /****************************************/
00095 
00096    void CLEDEntity::RemoveFromMedium() {
00097       try {
00098          GetMedium().RemoveEntity(*this);
00099          m_pcMedium = NULL;
00100          Disable();
00101       }
00102       catch(CARGoSException& ex) {
00103          THROW_ARGOSEXCEPTION_NESTED("Can't remove LED entity \"" << GetId() << "\" from medium.", ex);
00104       }
00105    }
00106 
00107    /****************************************/
00108    /****************************************/
00109       
00110    CLEDMedium& CLEDEntity::GetMedium() const {
00111       if(m_pcMedium == NULL) {
00112          THROW_ARGOSEXCEPTION("LED entity \"" << GetId() << "\" has no medium associated.");
00113       }
00114       return *m_pcMedium;
00115    }
00116 
00117    /****************************************/
00118    /****************************************/
00119 
00120    void CLEDEntitySpaceHashUpdater::operator()(CAbstractSpaceHash<CLEDEntity>& c_space_hash,
00121                                                CLEDEntity& c_element) {
00122       /* Discard LEDs switched off */
00123       if(c_element.GetColor() != CColor::BLACK) {
00124          /* Calculate the position of the LED in the space hash */
00125          c_space_hash.SpaceToHashTable(m_nI, m_nJ, m_nK, c_element.GetPosition());
00126          /* Update the corresponding cell */
00127          c_space_hash.UpdateCell(m_nI, m_nJ, m_nK, c_element);
00128       }
00129    }
00130 
00131    /****************************************/
00132    /****************************************/
00133 
00134    CLEDEntityGridUpdater::CLEDEntityGridUpdater(CGrid<CLEDEntity>& c_grid) :
00135       m_cGrid(c_grid) {}
00136 
00137    /****************************************/
00138    /****************************************/
00139 
00140    bool CLEDEntityGridUpdater::operator()(CLEDEntity& c_entity) {
00141       /* Discard LEDs switched off */
00142       if(c_entity.GetColor() != CColor::BLACK) {
00143          try {
00144             /* Calculate the position of the LED in the space hash */
00145             m_cGrid.PositionToCell(m_nI, m_nJ, m_nK, c_entity.GetPosition());
00146             /* Update the corresponding cell */
00147             m_cGrid.UpdateCell(m_nI, m_nJ, m_nK, c_entity);
00148          }
00149          catch(CARGoSException& ex) {
00150             THROW_ARGOSEXCEPTION_NESTED("While updating the LED grid for LED \"" << c_entity.GetContext() << c_entity.GetId() << "\"", ex);
00151          }
00152       }
00153       /* Continue with the other entities */
00154       return true;
00155    }
00156 
00157    /****************************************/
00158    /****************************************/
00159 
00160    REGISTER_STANDARD_SPACE_OPERATIONS_ON_ENTITY(CLEDEntity);
00161 
00162    /****************************************/
00163    /****************************************/
00164 
00165 }
```

---

Generated on 10 Jul 2018 for ARGoS by 
 1.6.1
